# Systemic and renal hemodynamic effects of intra-arterial radiocontrast

**DOI:** 10.1186/s40635-014-0032-z

**Published:** 2014-12-16

**Authors:** Paolo Calzavacca, Ken Ishikawa, Michael Bailey, Clive N May, Rinaldo Bellomo

**Affiliations:** Department of Anaesthesia and Intensive Care, Uboldo Hospital, Vi aUboldo 21, Cernusco sul Naviglio 20063 Milano, Italy; The Florey Institute of Neuroscience and Mental Health, The University of Melbourne, 156 Grattan St, Parkville 3010 Melbourne, Australia; Department of Intensive Care and Department of Medicine, Austin Hospital and The University of Melbourne, 145 Studley Rd, Heidelberg, 3084 Melbourne; Department of Pediatrics, Iwate Medical University, 19-1 Uchimaru, Morioka, Iwate Prefecture 020-0023 Japan; Australian and New Zealand Intensive Care Research Centre, Monash University, 99 Commercial Rd, Prahran 3004 Melbourne, Australia; Australian and New Zealand Intensive Care Research Centre, Monash University, Melbourne, Victoria Australia

**Keywords:** Radiocontrast agent, Angiography, Contrast-induced acute kidney injury, Renal vascular resistance, Vasoconstriction, Renal blood flow, Iodixanol

## Abstract

**Background:**

Decreased renal blood flow (RBF) and vasoconstriction are considered major mechanisms of contrast-induced acute kidney injury (CIAKI). To understand the severity and duration of such putative effects, we measured systemic and renal hemodynamics after intra-arterial radiocontrast administration. The subjects were six Merino ewes. The setting was a university-affiliated research institute. This is a randomized cross-over experimental study.

**Methods:**

Transit-time flow probes were implanted on the pulmonary and left renal arteries 2 weeks before experimentation. We simulated percutaneous coronary intervention by administering five intra-arterial boluses of 0.5 mL/kg saline (control) or radiocontrast (iodixanol) to a total of 2.5 mL/kg over 1 h. Cardiac output (CO), heart rate, mean arterial pressure (MAP), RBF, renal vascular conductance (RVC), urine output (UO), creatinine clearance (CrCl), and fractional excretion of sodium (FENa) were measured.

**Results:**

In the first 8 h after intra-arterial administration of radiocontrast, CO, total peripheral conductance (TPC), and heart rate (HR) increased compared with those after normal saline administration. Thereafter, CO and TPC were similar between the two groups, but HR remained higher with radiocontrast (*p* < 0.001). After a short (30 min) period of renal vasoconstriction with preserved RBF secondary to an associated increase in MAP, RBF and RVC showed an earlier and greater increase (vasodilatation) with radiocontrast (*p* < 0.001) and remained higher during the first 2 days. Radiocontrast initially increased urine output (*p* < 0.001) and FENa (*p* = 0.003). However, the overall daily urine output decreased in the radiocontrast-treated animals at 2 days (*p* < 0.001) and 3 days (*p* = 0.006). Creatinine clearance was not affected.

**Conclusions:**

In healthy animals, intra-arterial radiocontrast increased RBF, induced renal vasodilatation, and caused a delayed period of oliguria. Our findings suggest that sustained reduction in RBF and renal vasoconstriction may not occur in normal large mammals after intra-arterial radiocontrast administration.

## Background

Intra-arterial radiocontrast is used for coronary angiography and/or percutaneous coronary intervention (PCI) worldwide. In at-risk patients, radiocontrast can induce acute kidney injury (AKI). Such contrast-induced acute kidney injury (CIAKI) is one of the leading causes of AKI in hospitals [1-7]. CIAKI is independently associated with a higher mortality rate, hospital stay, and cost of care [3].

On the basis of limited experimental studies, two mechanisms have been proposed and are considered important to the development of CIAKI. One is a direct toxic effect of radiocontrast on renal tubular cells with ‘osmotic nephrosis’ [8-10], but the pathophysiological relevance of this is unclear because it has only been described *ex vivo* and it does not logically explain changes in glomerular filtration rate (GFR). The other possible mechanism is a decrease in renal blood flow (RBF) secondary to renal vasoconstriction. Human, dog, and small-animal studies have reported that intravenous infusion of contrast media significantly reduces total renal plasma flow [11-14]. However, in these studies, investigators administered amounts of contrast that exceed current practice, measured RBF with techniques of limited accuracy, and performed measurements only for a few hours after radiocontrast administration. The relevance of such findings to a condition which leads to peak GFR losses at 72 h is open to question. We recently used a validated methodology to measure systemic and renal hemodynamics directly, accurately, and over several days by implanted transit-time flow probes [15-17] and found that, contrary to expectation, *intravenous* contrast administration induced only short-lived renal vasoconstriction (first hour) followed by sustained (days) renal vasodilatation and increased RBF [18]. However, such observations may not apply to the most common trigger of CIAKI: intra-arterial boluses of radiocontrast given over a short time during PCI.

Accordingly, we studied the effect of intra-arterial radiocontrast in sheep while continuously measuring renal hemodynamics and repeatedly assessing renal function. We hypothesized that intra-arterial administration might, unlike intravenous administration, induce sustained renal vasoconstriction and, thereby, decrease GFR.

## Methods

### Animal preparation

We conducted a randomized cross-over study in six adult Merino ewes (weight 33 ± 1 kg). The animals were housed in individual metabolic cages, with free access to food and water. The experimental procedures were approved by the Animal Experimental Ethics Committee of the Florey Institute of Neuroscience under guidelines laid down by the National Health and Medical Research Council of Australia.

The animals underwent three sterile surgical procedures under general anesthesia at intervals of at least 2 weeks. Anesthesia was induced with intravenous sodium thiopentone (10 to 15 mg/kg) for intubation with an endotracheal tube (cuffed size 9). Maintenance of anesthesia was by means of oxygen/air/isoflurane (end-tidal isoflurane 1.5% to 2.0%). Fractional inspired oxygen was altered to maintain SatO_2_ above 97%, and ventilation was controlled to maintain end-tidal CO_2_ at approximately 35 mmHg. First, a bilateral carotid arterial loop was created to facilitate subsequent arterial cannulation. During the second procedure, a transit-time flow probe (Transonic Systems, Ithaca, NY, USA) was placed on the pulmonary artery through a left side fourth intercostal space thoracotomy. Finally, during the third procedure, a transit-time flow probe was placed on the left renal artery. The animals were allowed to recover at least 2 weeks from the last surgical procedure before the start of the experiments. In all operations, the animals were treated with intramuscular antibiotics (900 mg, Ilium Propen, procaine penicillin, Troy Laboratories Pty Ltd, Smithfield, NSW, Australia, or Mavlab, Slacks Creek, QLD, Australia) at the start of surgery and then for 2 days post-operatively. Post-surgical analgesia was maintained with intramuscular injection of flunixin meglumine (1 mg/kg) (Troy Laboratories Pty Ltd or Mavlab) at the start of surgery and then 8 and 24 h post-surgery.

The day before the experiment, a Tygon catheter (Cole-Parmer, Boronia, VIC, Australia; ID 1.0 mm, OD 1.5 mm, length 80 cm) was inserted into one carotid arterial loop to measure arterial pressure, to obtain blood samples, and to inject radiocontrast. The insertion of this catheter was performed under fluoroscopic guidance to confirm positioning of the catheter in the ascending aorta. Analog signals (mean arterial pressure (MAP), heart rate (HR), cardiac output (CO), and RBF) were collected on a computer using a customized data acquisition system (LabVIEW, National Instruments, Austin, TX, USA). The data were recorded at 100 Hz for 10 s every minute during experiments. Standard formulae were applied to calculate total peripheral conductance (TPC = CO/MAP) and renal vascular conductance (RVC = RBF/MAP).

Urine output (UO) was collected hourly through an automated urine fraction collector at hourly intervals for 5 days (2 days baseline, day 0, and 2 days post-intervention). Two-hour creatinine clearance (CrCl) and fractional excretion of sodium (FENa) were calculated according to standard formulae: CrCl = (U_Creat_ × UO)/(P_Creat_/time(min)), where U_Creat_ is urine creatinine, P_Creat_ plasma creatinine, and UO urine volume in 2 h; FENa = *P*_Na_/*U*_Na_ × CrCl × 100, where *P*_Na_ is plasma concentration and *U*_Na_ urine concentration of sodium.

### Experimental protocol

Baseline hemodynamic and UO measurements were collected for 48 h prior to the administration of control fluid (normal saline) or the radiocontrast agent (iodixanol, Visipaque™, GE Healthcare, Milwaukee, WI, USA). To replicate a coronary angiogram study (PCI), we observed several PCIs at our institution and replicated practice in our model. Thus, normal saline or iodixanol was infused intra-arterially as five boluses of 0.5 mL/kg at 12-min intervals. Blood samples were taken before the start and then at 2, 4, 8, 24, 48, and 72 h after the injections. Urine was collected hourly throughout the experiment from the bladder catheter and sampled in 2-h lots at the predefined time points for creatinine clearance and fractional excretion of urea measurements up to the third day after the administration of control or radiocontrast. Bladder catheters were then removed. The hemodynamic parameters were followed for a total of 5 days after saline or radiocontrast administration. The animals had free access to food and water with food given at 5 p.m. every day. However, the day of the injection, fluid and water were restricted from 8 a.m. to 4 p.m. to avoid confounders on urine output and hemodynamic variables. Daily water intake was also recorded for every animal.

### Statistical analysis

All data are presented as means ± standard error of means (SEM) or geometric means (95% confidence interval) as appropriate. For baseline values, averages of the 48 h of baseline are presented. Hour-by-hour values for the first 8 h and daily averages up to the fifth day were compared with the average of baseline values by analysis of variance. To adjust for multiple comparisons, *p* < 0.01 was used to indicate significance. Statistical analysis was performed using SAS version 9.2 (SAS Institute Inc., Cary, NC, USA). All variables were assessed for normality and log-transformed where appropriate.

Mixed linear modeling was performed with each sheep treated as a random effect. Main effects were fitted for time and treatment with an interaction between time and treatment used to determine if treatments behaved differently over time. Specific time point comparisons were performed using *post hoc* pairwise *t*-tests. To account for multiple comparisons, a reduced *p* value of 0.01 was considered to be statistically significant.

## Results

### Systemic hemodynamics

**Figure 1:**
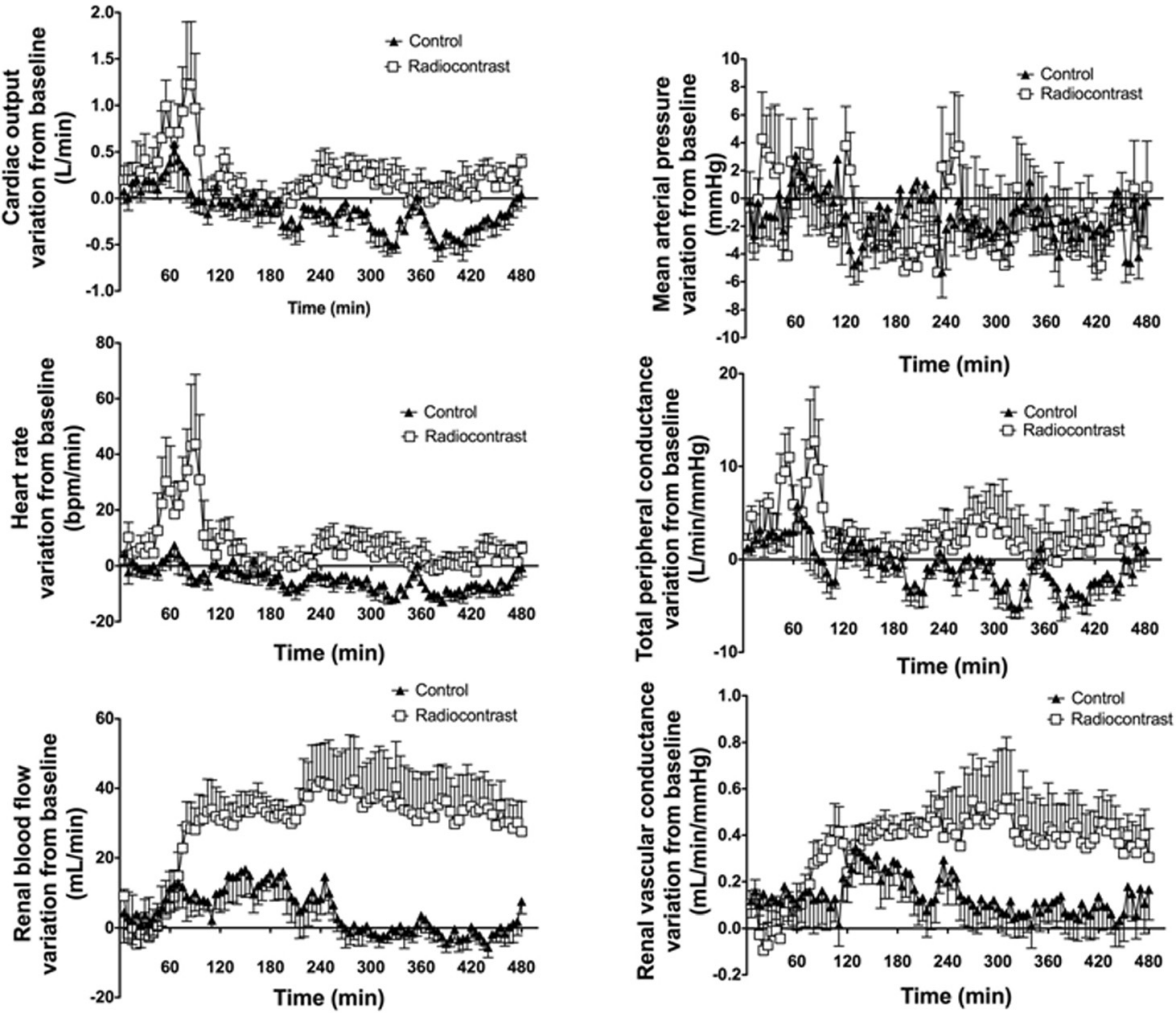
**Hemodynamic changes in the first 8 h after radiocontrast administration.** Changes in systemic and renal hemodynamics from baseline after an intra-arterial bolus of radiocontrast agent or saline. Values are mean ± SEM at 5-min intervals.

**Figure 2:**
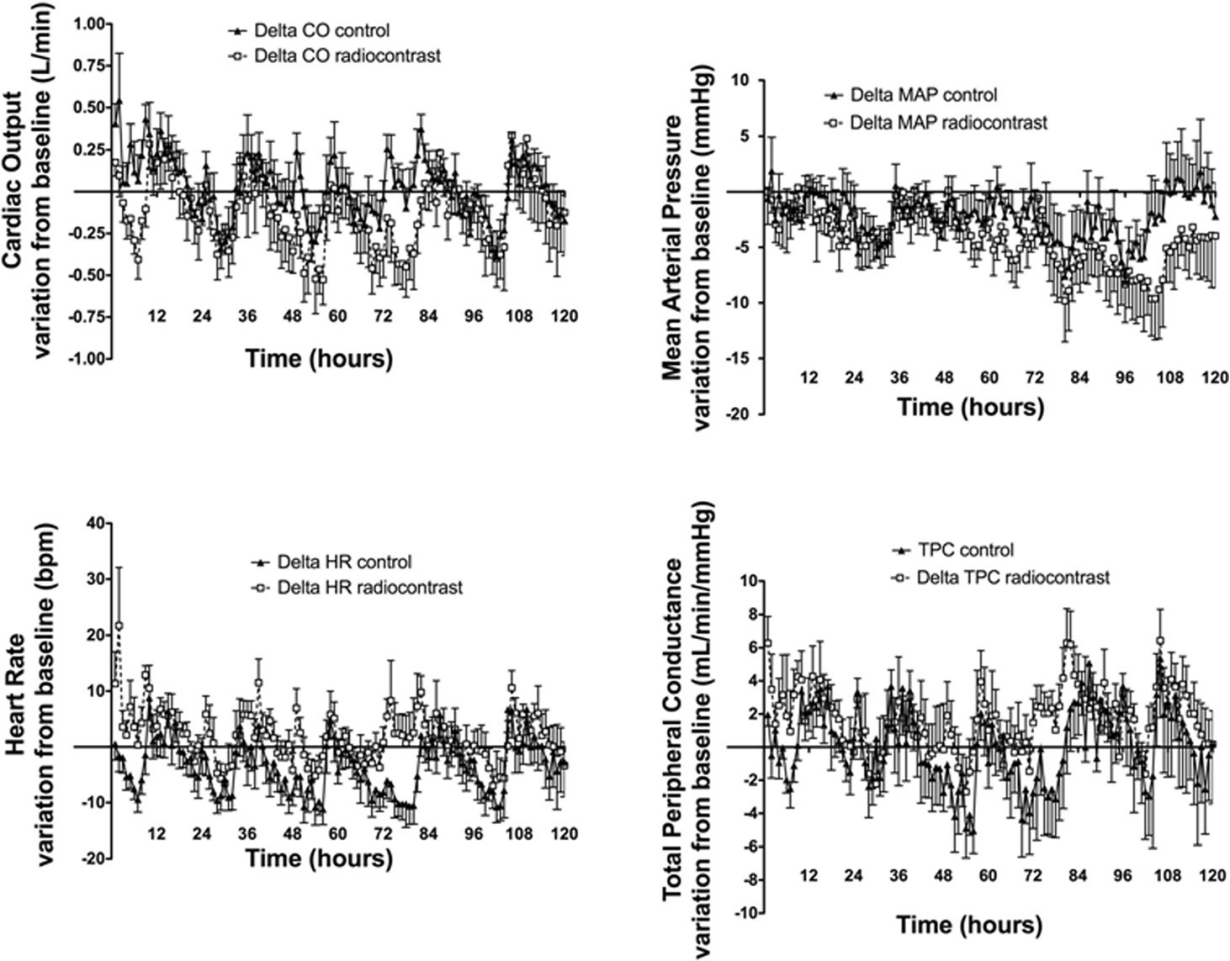
**Changes in systemic hemodynamics over a 5-day period of observation.** Changes in systemic and renal hemodynamics from baseline after an intra-arterial bolus of radiocontrast agent or saline. Values are mean ± SEM at hourly intervals.

In the first 8 h, intra-arterial administration of radiocontrast increased CO, TPC, and HR more than that of normal saline (Table 1 and Figure 1) without any difference in MAP. Daily changes in CO and TPC were not different between the two groups (*p* = 0.10 and *p* = 0.30, respectively, for CO and TPC) (Table 2 and Figure 2). MAP was higher at baseline in the radiocontrast-treated animals and remained higher with radiocontrast during the following 5 days, with significant differences (*p* < 0.001) between groups (Table 2). HR was also higher with radiocontrast (Table 2). The differences between the groups, although statistically significant, were small and occurred with full preservation of the normal circadian rhythm (Figure 2).

**Table 1:** **Haemodynamic values at baseline and during the first 8 hours after arterial radiocontrast**

	**GROUP**	**Baseline**	**1** ^**st**^ **hour**	**2** ^**nd**^ **hour**	**3** ^**rd**^ **hour**	**4** ^**th**^ **hour**	**5** ^**th**^ **hour**	**6** ^**th**^ **hour**	**7** ^**th**^ **hour**	**8** ^**th**^ **hour**
CO (L/min)	Saline	3.52 (±0.26)*	3.70 (±0.27)°	3.65 (±0.27)	3.46 (±0.27)*	3.35 (±0.27)°	3.36 (±0.27)°	3.23 (±0.27)°	3.12 (±0.27)°	3.35 (±0.27)°
	Contrast	3.25 (±0.26)*	3.66 (±0.27)°	3.80 (±0.27)°	3.31 (±0.27)*	3.30 (±0.27)	3.53 (±0.27)°	3.37 (±0.27)	3.32 (±0.27)	3.47 (±0.27)°
HR (bpm)	Saline	65 (±4)	66 (±5)*	65 (±5)*	64 (±5)	60 (±5)°*	60 (±5)°*	58 (±5)°*	56 (±5)°*	60 (±5)°*
	Contrast	64 (±4)	76 (±5)°*	86 (±5)°*	68 (±5)	67 (±5)*	72 (±5)°*	68 (±5)*	65 (±5)*	69 (±5)°*
MAP (mmHg)	Saline	88 (±2)*	87 (±2)	88 (±2)	85 (±2)°	87 (±2)	86 (±2)	87 (±2)	85 (±2)°	86 (±2)
	Contrast	95 (±2)*	95 (±2)	95 (±2)	92 (±2)°	91 (±2)°	94 (±2)	93 (±2)	92 (±2)°	93 (±2)
TPC (mL/min/mmHg)	Saline	39.8 (±3.0)*	42.2 (±3.0)°*	41.0 (±3.0)	40.4 (±3.0)*	38.3 (±3.0)	38.9 (±3.0)	37.1 (±3.0)°	36.3 (±3.0)°	38.8 (±3.0)
	Contrast	35.0 (±3.0)*	39.9 (±3.0)°*	40.3 (±3.0)°	36.3 (±3.0)*	36.7 (±3.0)°	38.4 (±3.0)°	36.5 (±3.0)	36.3 (±3.0)	37.9 (±3.0)°
RBF (mL/min)	Saline	167 (±19)*	171 (±19)*	176 (±19)°	181 (±19)°*	177 (±19)°*	170 (±19)*	166 (±19)*	165 (±19)*	166 (±19)*
	Contrast	156 (±19)*	157 (±19)*	183 (±19)°	189 (±19)°*	191 (±19)°*	195 (±19)°*	191 (±19)°*	190 (±19)°*	188 (±19)°*
RVC (mL/min/mmHg)	Saline	1.83 (±0.19)*	1.96 (±0.19)°*	1.96 (±0.19)°	2.11 (±0.19)°	2.00 (±0.19)°*	1.96 (±0.19)°*	1.90 (±0.19)°*	1.92 (±0.19)°*	1.93 (±0.19)°*
	Contrast	1.66 (±0.19)*	1.68 (±0.19)*	1.95 (±0.19)°	2.05 (±0.19)°	2.10 (±0.19)°*	2.12 (±0.19)°*	2.09 (±0.19)°*	2.06 (±0.19)°*	2.04 (±0.19)°*
UO (mL/h)	Saline	12 (±8)	30 (±10)*	27 (±10)*	27 (±10)	23 (±10)	25 (±10)	21 (±10)	24 (±10)	25 (±10)
	Contrast	17 (±8)	66 (±10)°*	70 (±10)°*	30 (±10)	30 (±10)	16 (±10)	17 (±10)	10 (±10)	15 (±10)

°: p≤0.01 for within group time comparison with baseline; *: p≤0.01 for between-group comparison. Note: UO baseline: average of last 2 hours of baseline monitoring.

**Table 2:** **Haemodynamic values during the first 5 days after radio-contrast injection**

	**GROUP**	**Baseline**	**1** ^**st**^ **day**	**2** ^**nd**^ **day**	**3** ^**rd**^ **day**	**4** ^**th**^ **day**	**5** ^**th**^ **day**
CO (L/min)	Saline	3.52 (±0.26)*	3.52 (0.30)	3.41 (0.30)°*	3.29 (0.30)°	3.40 (0.30)°	3.46 (0.30)*
	Contrast	3.25 (±0.26)*	3.42 (0.30)°	3.24 (0.30)*	3.18 (0.30)	3.31 (0.30)	3.21 (0.30)*
HR (bpm)	Saline	65 (±4)	64 (±4)*	61 (±4)°*	60 (±4)°*	62 (±4)°*	63 (±4)
	Contrast	64 (±4)	69(±4)°*	65 (±4)*	64 (±4)*	67 (±4)*	65 (±4)
MAP (mmHg)	Saline	88 (±2)*	87 (±1)*	85 (±1)°*	86 (±1)*	84 (±1)°*	86 (±1)*
	Contrast	95 (±2)*	93 (±1)°*	92 (±1)°*	91 (±1)°*	89 (±1)°*	89 (±1)°*
TPC (mL/min/mmHg)	Saline	39.8 (±3.0)*	40.5 (±3.6)*	39.9 (±3.6)*	38.1 (±3.6)°*	40.6 (±3.6)*	40.2 (±3.6)*
	Contrast	35.0 (±3.0)*	37.5 (±3.6)°*	35.6 (±3.6)*	35.3 (±3.6)*	37.8 (±3.6)°*	36.9 (±3.6)°*
RBF (mL/min)	Saline	167 (±19)*	171 (±18)*	166 (±18)	163 (±18)*	166 (±18)*	174 (±18)*
	Contrast	156 (±19)*	181 (±18)°*	163 (±18)°	153 (±18)*	155 (±18)*	150 (±18)*
RVC (mL/min/mmHg)	Saline	1.83 (±0.19)*	1.93 (±0.18)°	1.94 (±0.18)°*	1.88 (±0.18)°*	1.97 (±0.18)°*	2.00 (±0.18)°*
	Contrast	1.66 (±0.19)*	1.97 (±0.18)°	1.78 (±0.18)°*	1.69 (±0.18)°*	1.75 (±0.18)*	1.72 (±0.18)°*
UO (mL/h)	Saline	20 (±3)	24 (±3)	18 (±3)	16 (±3)		
	Contrast	20 (±3)	22 (±3)	14 (±3)°	16 (±3)°		
Daily UO (mL/24 hours)	Saline	380 (262;650)*	535 (341;813)	441 (344;532)*	386 (276;512)		
	Contrast	543 (162;924)*	529 (534;295)	349 (257;427)*	329 (196;596)*		

°: p≤0.01 for within group time comparison with baseline; *: p≤0.01 for between-group comparison.

UO: urinary output during that hour; daily UO: average of urinary output over24 hours. Bladder catheter removed after day 3.

### Renal hemodynamics

**Figure 3:**
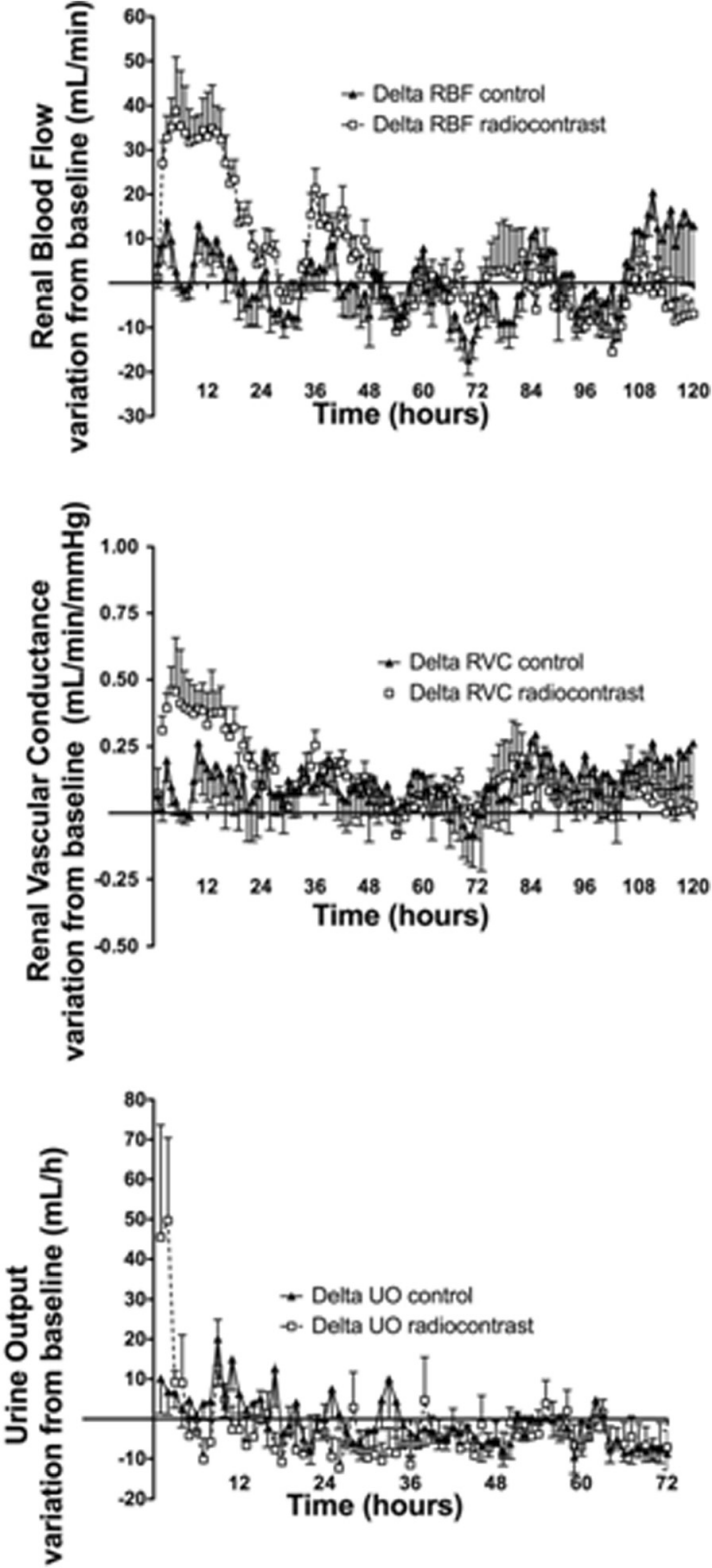
**Changes in renal hemodynamics over a 5-day period of observation.** Changes in systemic and renal hemodynamics from baseline after an intra-arterial bolus of radiocontrast agent or saline. Values are mean ± SEM at hourly intervals.

After a short (30 min) period of renal vasoconstriction with preserved RBF secondary to an associated increase in MAP (Figure 1), in the first 8 h (Table 1 and Figure 1), RBF and RVC showed an earlier and greater increase in the radiocontrast-treated animals (*p* < 0.001) and both RBF and RVC remained higher during the following 2 days (Table 2 and Figure 3). By day 3, RBF and RVC had returned to baseline values. The circadian rhythms of RBF and RVC were preserved.

### Renal function

**Figure 4:**
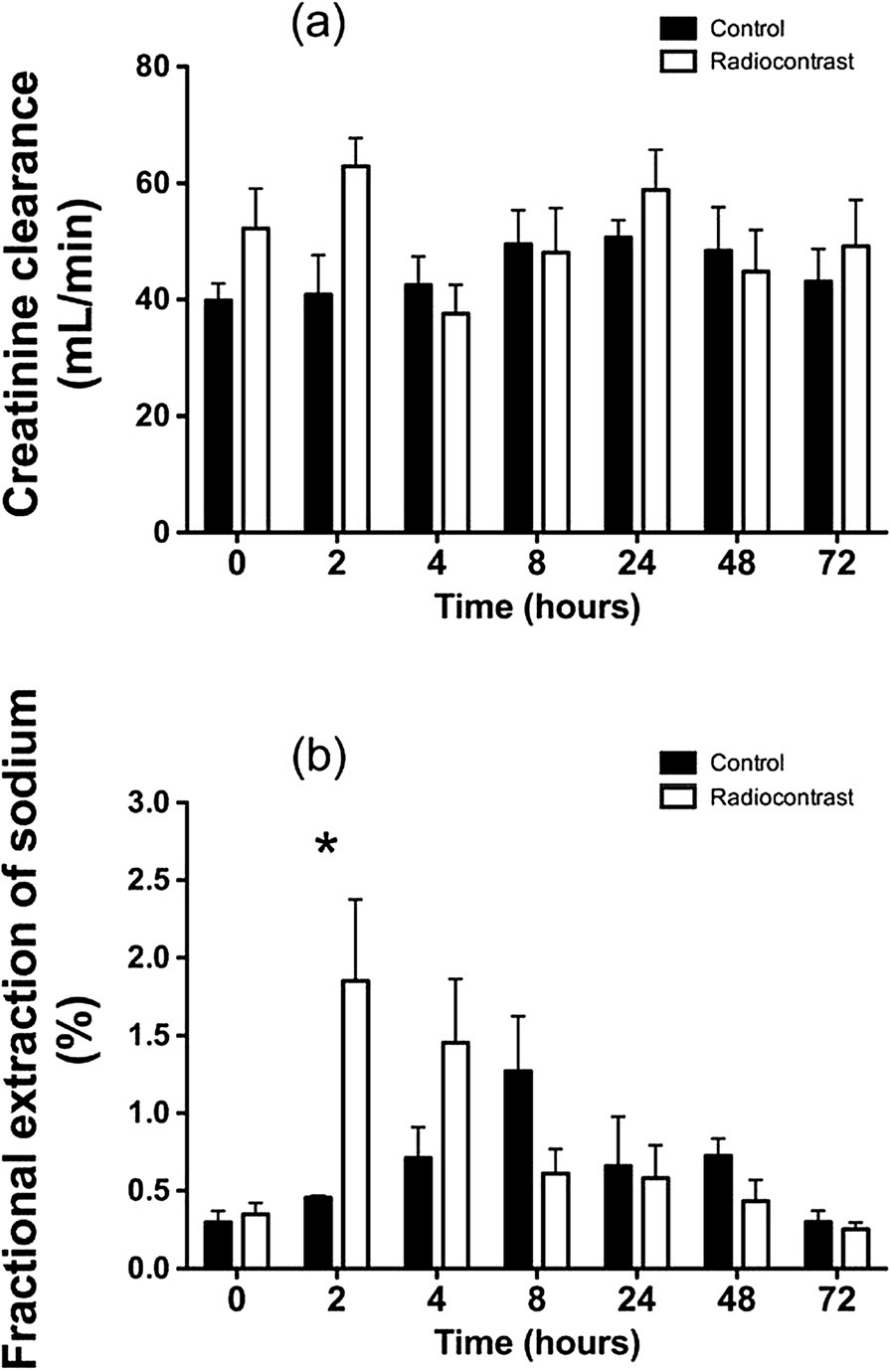
**Renal functional changes after radiocontrast administration. (a)** Creatinine clearance and **(b)** fractional excretion of sodium after an intra-arterial bolus of radiocontrast agent or saline. Values are mean ± SEM. Asterisk indicates *p* ≤ 0.01 for between-group comparison.

Radiocontrast increased urine output at the first and the second hour (both *p* < 0.001 compared to baseline) (Figure 3) and FENa at 2 h (*p* = 0.003 for comparison with baseline) (Figure 4). Normal saline did not affect urine output, creatinine clearance, or fractional excretion of sodium. Creatinine clearance was not changed at any time point by radiocontrast administration; however, the overall daily urine output decreased in the radiocontrast-treated animals after 2 days (*p* < 0.001) and 3 days (*p* = 0.006) (Table 2).

## Discussion

### Key findings

We performed an experimental study in conscious large animals to assess the systemic and renal hemodynamic effects of repeated intra-arterial boluses of radiocontrast, simulating PCI-associated contrast administration. Measurements were taken over 5 days following administration of contrast, the period when CIAKI typically becomes clinically manifest. Intra-arterial radiocontrast induced an early and short-lived (hours) increase in heart rate and cardiac output with peripheral vasodilatation. Contrary to our hypothesis, we found that radiocontrast caused a large increase in renal blood flow and renal vasodilatation in the first 8 h, which returned to control over 48 h. Functionally, radiocontrast did not affect creatinine clearance, but caused an early (first 2 h) increase in urine output and FENa followed by a delayed decrease in urine output by days 2 and 3.

### Relationship to previous studies

Our experimental findings are unique because of the detailed and prolonged measurement of RBF with highly accurate technology. No such extended studies of the systemic and renal hemodynamic effects of intra-arterial radiocontrast administration have been performed. Our findings are at odds with some previous studies which demonstrate vasoconstriction after contrast medium administration [11-14]. They are, however, in concordance with other work by our group, which found that intravenous radiocontrast induced a quantitatively dominant and prolonged renal vasodilatation lasting at least 72 h after treatment. They are also in concordance with the only other study that performed some limited extended monitoring of RBF after radiocontrast administration [19].

If radiocontrast injection also induced vasodilatation and global hyperemia in man, it might seem counterintuitive that decreased GFR and oliguria would follow, as seen with CIAKI in at-risk patients. However, glomerular capillary filtration pressure is dependent on the relationship between afferent and efferent arteriolar tone. If, for example, afferent vasodilatation was associated with greater efferent vasodilatation (as might be the case with angiotensin-converting enzyme inhibitors), then an increase in renal blood flow (hyperemia) would occur together with a decrease in intraglomerular capillary pressure, oliguria (as seen in our animals), and a decrease in GFR (not seen in our healthy animals or in previously healthy patients). This mechanism may be responsible for the decreased GFR of radiocontrast exposure. Studies using selective efferent arteriolar vasoconstrictors may help clarify the role of this mechanism in the pathogenesis of the observed vasodilatation.

#### Implications of study findings

Our findings do not support the view that the functional changes induced by intra-arterial radiocontrast administration are explained by reduced RBF and/or renal vasoconstriction as is currently reported and published [20]. They imply that efforts to prevent CIAKI by administering agents known to induce vasodilatation (low-dose dopamine and/or fenoldopam) are unlikely to be successful and that vasopressor agents that preferentially increase resistance in the efferent arteriole [19,20] may be more logical.

#### Strengths and limitations

Our study has several strengths. First, we measured RBF and renal vascular conductance continuously with highly accurate technology, every minute for an extended period of observation. Second, we created a model which simulated the observed practice of intra-arterial radiocontrast administration as delivered in the cardiac catheterization laboratory of a tertiary invasive cardiology unit, making our findings highly relevant at a clinical level. Third, we combined systemic and renal hemodynamics with some measures of renal function.

However, our experimental study also has several limitations. We studied a specific radiocontrast agent and our findings may not apply to different agents. We studied a specific species which may not be representative of effects in other animals, including man. We used normal animals and our model did not induce a decrease in GFR. Thus, it is uncertain how these findings may apply in the setting of contrast-induced nephropathy or in humans where age and comorbidities profoundly influence renal functional outcomes. We were unable to assess tubular injury, which may be an important mechanism leading to subsequent loss of GFR. We did not measure GFR with sophisticated and sensitive techniques. Thus, changes in GFR may have occurred and have been missed. However, the clinical significance of such changes (if present) would remain unclear. We did not measure biomarkers of tubular injury [21], but our research focus was on the systemic and renal hemodynamic effects of radiocontrast and the relevance of such biomarkers remains unclear [22,23]. We did not obtain biopsies to ascertain the degree of apoptosis or histological injury. However, in order to conduct the experiments to 5 days, we were not able to perform *post-mortem* studies and did not want to affect hemodynamic measurements by any damage that biopsy might have induced. Moreover, our measurements apply to global renal blood flow and do not provide information on changes in the renal microvasculature which might have diverted blood to either the cortex or the medulla and caused medullary hypoxia as shown in animal models by blood oxygen level-dependent (BOLD) MRI-based techniques [24-26]. We also acknowledge that there is substantial evidence that contrast agents decrease medullary perfusion and oxygenation. For instance, BOLD MRI studies showed declining medullary oxygenation in healthy individuals [27]; contrast media lead to pimonidazole immunostaining and stabilize medullary HIF to a moderate extent, even without COX or NOS inactivation [28], particularly in the presence of predisposing factors, such as diabetes [29]. Furthermore, at least one study found tubuloglomerular feedback (TGF) activation when contrast media were flushed through the medullary thick ascending loop of Henle/macula densa [30], providing evidence for a tubular process causing loss of GFR. Our study does not address any of these possible mechanisms of injury. Finally, data such as tissue hypoxia [31], declining medullary flows [32], or urine NGAL release [33] suggest that renal medullary hypoperfusion and parenchymal injury conceivably take place at the very early period after the injection of radiocontrast. It remains uncertain, however, whether such a large body of observed pathophysiological events truly contributes to AKI or represents a constellation of epiphenomena.

## Conclusions

In an experimental study extended over 5 days, we found that intra-arterial radiocontrast increased renal blood flow and induced renal vasodilatation. This effect was marked in the first 8 h and persisted for approximately 48 h. Our findings suggest that sustained reduction in RBF and renal vasoconstriction may not occur in normal large mammals after intra-arterial radiocontrast administration.
